# Functional Stimuli-Responsive Gels: Hydrogels and Microgels

**DOI:** 10.3390/gels4020054

**Published:** 2018-06-12

**Authors:** Coro Echeverria, Susete N. Fernandes, Maria H. Godinho, João Paulo Borges, Paula I. P. Soares

**Affiliations:** 1 Instituto de Ciencia y Tecnología de Polímeros, ICTP-CSIC, Calle Juan de la Cierva 3, Madrid 28006, Spain; 2 I3N/CENIMAT, Department of Materials Science, Faculty of Science and Technology, Universidade NOVA de Lisboa, Campus de Caparica, Caparica 2829-516, Portugal; sm.fernandes@fct.unl.pt (S.N.F.); mhg@fct.unl.pt (M.H.G.); jpb@fct.unl.pt (J.P.B.)

**Keywords:** functional gels, hydrogels, microgels, hybrid microgels, stimuli-responsive, shape memory hydrogels, self-healing gels

## Abstract

One strategy that has gained much attention in the last decades is the understanding and further mimicking of structures and behaviours found in nature, as inspiration to develop materials with additional functionalities. This review presents recent advances in stimuli-responsive gels with emphasis on functional hydrogels and microgels. The first part of the review highlights the high impact of stimuli-responsive hydrogels in materials science. From macro to micro scale, the review also collects the most recent studies on the preparation of hybrid polymeric microgels composed of a nanoparticle (able to respond to external stimuli), encapsulated or grown into a stimuli-responsive matrix (microgel). This combination gave rise to interesting multi-responsive functional microgels and paved a new path for the preparation of multi-stimuli “smart” systems. Finally, special attention is focused on a new generation of functional stimuli-responsive polymer hydrogels able to self-shape (shape-memory) and/or self-repair. This last functionality could be considered as the closing loop for smart polymeric gels.

## 1. Introduction

Gels have pervaded our everyday life in a variety of forms. The wet soft solids that we encounter in the form of commercial products such as soap, shampoo, toothpaste, hair gel and other cosmetics, as well as contact lenses and gel pens, etc., are all gels derived from polymeric compounds. Polymer gels have been known for centuries and used for application in fields as diverse as food, medicine, materials science, cosmetics, pharmacology, and sanitation, among others. In general, gels are viscoelastic solid-like materials comprised of an elastic cross-linked network and a solvent, which is the major component. The solid-like appearance of a gel is a result of the entrapment and adhesion of the liquid in the large surface area solid three-dimensional (3D) matrix [1].

**Figure 1:**
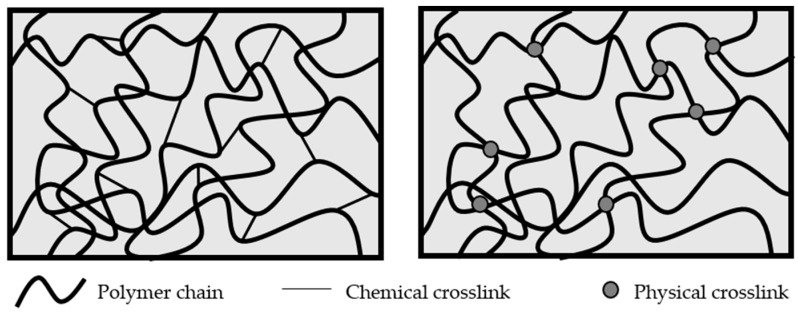
Schematic representation of polymer gels formed by chemical crosslinks (**left** image) or physical crosslinks (**right** image) [2].

Pierre-Gilles de Gennes during his Nobel lecture in 1991 recognized the place of gels among the broad category of ‘‘soft matter’’. Focusing on the phenomenological characteristics, polymer gels are 3D networks swollen by a large amount of solvent. Solid-like gels are characterized by: (i) the absence of an equilibrium modulus; (ii) the presence of a storage modulus, G’(u), which exhibits a pronounced plateau extending to times at least of the order of seconds; and (iii) the presence of a loss modulus, G’’(u), which is considerably smaller than the storage modulus in the plateau region. The presence of liquid-like behavior on molecular length scales combined with solid-like macroscopic properties makes them very unique systems. Polymer gels are classified as chemical and physical gels, depending on the nature of crosslinks (Figure 1) [2].

**Figure 2:**
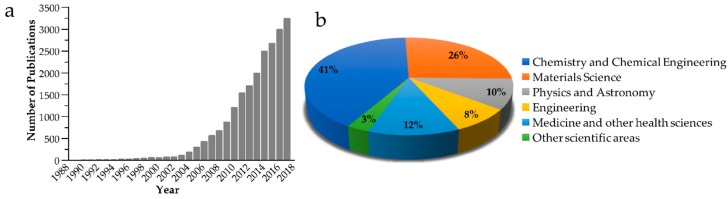
(**a**) Number of publications per year and (**b**) publications by subject featuring stimuli-responsive gel. Databased used for the bibliographic analysis: Scopus^®^ Elsevier, Amsterdam, The Netherlands consulted on the 2nd of May 2018.

Considering the current needs in the field of materials science, the strategy that has gained attention in the last decades is the understanding and further mimicking of structures and behaviours found in nature in order to develop materials with additional functionalities. These materials will have the ability to respond to several stimuli and could be used for biomedical purposes or in everyday life applications. In this context, the so-called “smart” or responsive polymers are becoming increasingly important. This kind of polymer is able to transform external stimuli into shape change or movement by taking advantage of the surrounding environment. The interest in the stimuli-responsive polymers has continued over many decades and it is still a hot topic in materials science. Indeed, researchers’ interest in the ability that this kind of gels present when subjected to external stimuli can be seen in the literature since the early 1990’s (Figure 2a) and is transversal to several scientific subjects (Figure 2b). In this regard, polymer gels have a special place in the field of stimuli responsive systems, and have attracted much attention due to their composition, versatility, and wide variety of potential applications, in particular in the biomedical field. However, we can also find its use within fields as catalysis, mechanical, chemical or optical sensors, or actuators, among others.

The nature of the stimulus can be classified as physical, chemical or biological [3]. Physical stimuli influence the systems molecular interactions, while the last two types of stimuli directly affect the interactions between the polymer chains (intramolecular) or with other constituents of the system. However, in both cases, a phase transition is observed. Gels can be divided into macrogels, microgels and nanogels and can be assembled to give rise to thin-films, brushes, membranes, colloidal particles, foams, among others, as is well explained by Stuart et al. [4].

In this review, we focus attention on recent advances found within the field of stimuli responsive functional polymeric gels. In the first section of the review, we will devote our attention to earlier (last year) developments on stimuli-responsive polymer hydrogels. Some examples are presented where stimuli as temperature, pH, light and redox, or analyte-responsive hydrogels and 3D printing applications are highlighted. The second section of the review will focus on the most recent studies regarding multifunctional stimuli-responsive microgels, in particular hybrid microgels systems. Special attention will be paid to (multi)responsiveness induced by inorganic nanoparticles incorporated within the microgel matrix, such as, metallic, magnetic, or carbonaceus particles. Finally, the last part of this review aims to highlight the new generation of functional stimuli-responsive polymer hydrogels—self-shape (shape memory) or self-healing hydrogels, as a step-forward in the field of “smart” materials.

## 2. Stimuli-Responsive Functional Hydrogels

Hydrogel (water containing gels) can be defined as a polymeric 3D network insoluble in water. This polymeric network is composed of a polar and hydrophilic natural and/or synthetic polymer that is physically or chemically crosslinked and can uptake a large amount of water. If within the polymer hydrogel network, one element can act as a receptor unit or is spectroscopically active, this gel is called a stimuli-responsive or smart material [1,2]. The effect that an external stimulus induces in the hydrogel network can be observed/measured often on a macroscopic scale, in different forms as change in shape and size, changes in its optical, wettability, electric and mechanical properties [4].

Several reviews regarding the production and application of hydrogels can be found in literature in recent years. These documents can be generic [5,6,7] or divided by the type of polymer (as for instance natural polymers [8], cellulose nanocrystals [9], DNA [10]), mechanical properties [11], applications (drug delivery [12,13], drug delivery and tissue engineering [14]), fabrication process and for instance a combination of type of polymer and application ((poly(*N*-isopropylacrylamide) (PNIPAAm) and biomedical applications [15], biomolecules in medicine [16], sensing devices and drug delivery [17,18], sensing molecular targets [19], PNIPAAm as photonic sensor [20]). It is important to note that due to the high number of publications on a specific sub-topic (for instance the use of PNIPAAm within the hydrogels), only a small selection of the most recent articles will be presented. With this selection, the authors intend to show the reader a broader view of this type of systems, its properties, and capabilities.

### 2.1. Thermoresponsive Hydrogels

Temperature is one of the most employed stimuli within these 3D systems since it is easier to regulate and can be used for in vitro and in vivo testing. The temperature point at which a response is observed is called the critical temperature (Tcr) or volume phase transition temperature (TvPT). At this point, a phase change occurs between the polymer and solvent within the system (depending on the system composition) and the phase is enhanced in one of the constituents. If this phase separation occurs above the critical temperature, the polymer exhibits a lower critical solution temperature (LCST) behavior; if this transition is observed below Tcr, an upper critical solution temperature (UCST) is shown by the polymer. Systems with LCST behavior are the most studied.

Polymer gels containing PNIPAAm are amongst the most investigated since its lower LCST in water (~32 °C) favors its potential use in such an important area as biomedicine. Micro/nanogels and hydrogels of PNIPAAm contract upon heating and swell with water upon cooling, and this transition is fully reversible [21,22]. The study of this polymer is so important that a constant development in the experimental reaction conditions can be found in the literature. Keeping this in mind, Tang and co-workers [23] recently synthesized hydrophobic crosslink, 4,4′-dihydroxybiphenyl diacrylate that enables the production of PNIPAAm hydrogels through free radical polymerization. With this system, the authors were able to decrease hydrogels’ LCST (up to 4.3 °C when compared with the poly(ethylene glycol) (PEG) 400 dimethacrylate-PNIPAAm hydrogel control), while maintaining a reversible thermal-responsive.

Nevertheless, the interest in PNIPAAm-based hydrogel has attracted scientists in other areas as shown recently by Kim et al. [24]. The authors produced a nature-inspired multi-functional hydrogel based in PNIPAAm and poly(pyrrole) (PPY), which combines the thermoresponsive nature of the first polymer with the conductive nature of the second one. To make this system, the authors prepare a photo-crosslinked thermoresponsive PNIPAAm membrane with specific patterns, in accordance with its envisioned use. This membrane was then deswelled and subsequently swelled with PPY monomer solution. The conductive layer was finally formed through in situ polymerization by addition of ammonium persulfate and phytic acid solution. This hydrogel system presents a controllable thermoresponsive deformability and electric conductivity. Moreover, upon heating, the hydrogel acts as a filtration membrane for the separation of proteins from water, mimicking the properties observed on functional features of plants such as bending of the pulvinus in *M. pudica* (genus Mimosa), the gating of stomata in leaf, and the selective filtering of the cellular membrane.

More recently, D’Eramo et al. [25] using a photo-patterning technique simultaneously grafted, patterned and crosslinked PNIPAAm onto plane substrate. With this biotechnologic approach that consists mainly of three steps (photo-patterning of hydrogel films, thiol-modification of substrates and closure of the device with an optical adhesive or silicone polymer), the authors were able to produce a microfluidic valve. This microvalve is controlled with temperature, i.e., above the LCST the valve is open. The authors were able to generate a platform of 7800 cages that were efficient in capturing and releasing solutes on-demand with 100-millisecond-scale response times. With these systems, the authors also showed that this hydrogel technology could be used in single-cell trapping/release and DNA amplification. For example, it was shown as an effective amplification of the human gene synaptojanin-1, linked with Parkinson disease.

**Figure 3:**
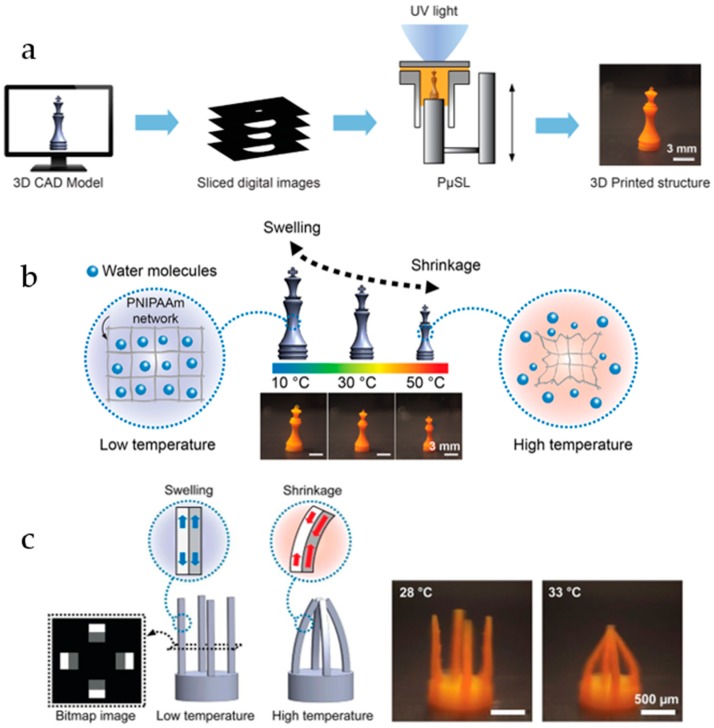
3D printing of PNIPAAm thermo-responsive hydrogel: (**a**) schematic illustrating of the printing process that goes from the drawing of the 3D model with computer-aided-design (CAD) software, that is then digitally sliced into a series of cross-sectional images. The 3D structure is obtained with PμSL by curing the liquid resin with a series of masks (derived from each slice) optically patterned with ultra-violet light; (**b**) schematic drawing of the temperature response of the 3D printed hydrogel with a phase change when the temperature decreases or increases, observed by swelling and shrinkage, respectively. In the middle (photographs), one can see this effect on the printed 3D structure (at T of 10, 20 and 50 °C); (**c**) in this sequence of sketches and pictures one can see, from left to right, a slice of the gripper with two arms and two different levels of gray to be able to produce a 4 arm 3D structure. The arms of this hydrogel bend when the temperature is higher than the temperature transition, depicted in the central region, and photos of the bending responsive observed when the 3D gel is placed at 33 °C (Figure adapted under the terms of the CC BY 4.0 License) [26].

Exploration of new methods to produce PNIPAAm-based hydrogels is constant as shown by the recent work of Han et al. [26]. In this essay, the authors were able to obtain 3D PNIPAAm micro-structures, using a high-resolution projection micro-stereolithography (PμSL), as schematic presented in Figure 3a. Materials with different shapes (Figure 3b,c) were prepared and could present deformation upon pre-programmed thermal-stimulus. With temperature, hydrogel’s swelling was tunable by changes on the crosslinker to monomer ratio and shrinking controlled by monomer concentration. The authors, by controlling the printing parameter, were also able to produce materials with an anisotropic swelling behavior. Using these features, the authors created a structure with layers that present different swelling ratios above the hydrogel’s temperature transition (Figure 3c). The different swelling ratios lead to a strain in the structure and a bending deformation can be observed as the temperature is cycled.

Grafting of PNIPAAm in a natural polymer backbone, as in the chitosan (CS), has also attracted attention from the scientific community due to their biocompatibility, low toxicity and their high content of functional groups. Luckanagul et al. [27] were able to prepare nanogels with control efficiency drug delivery. It was also possible to control the drug loading capacity of the gels by changing the degree of substitution of PNIPAAm in CS.

Another interesting example of thermo-responsive hydrogel is the approach proposed by Fang and co-workers [28]. In this essay, PNIPAAm hydrogel was coated with a layer of cell-adherent arginine-glycine-aspartate (RGD) peptides that successfully rupture cancer cells attached to the hydrogel surface. The authors explored the physical force of the expanding stimuli-responsive hydrogel to obtain such an interesting result with this simple approach.

One can also find another type of thermoresponsive hydrogel polymers as the biocompatible, fully synthetic polymer polyisocyanopeptide [29], which exhibits a LCST behavior. Zimoch and co-workers [30] were able to show that the synthetic GLY-ARG-GLY-ASP-SER peptide conjugated to the polymer backbone as a hydrogel was able to support complex cell culture, such as adipogenic differentiation and formation of blood capillaries. The thermoresponsive behavior of this system was able to induce a quick and easy retraction of viable cells from the hydrogel structure, eliminating the need for enzymes or other time-consuming procedures.

### 2.2. pH-Responsive Hydrogels

If a in a polymer backbone ionizable side chains (groups that can donate and accept ions) are grafted, a system that responds to external pH changes is developed (normally observed when pH is raised above the pK_a_ of the gel). These systems can form polyelectrolytes with water and are classified as anionic, cationic or amphiphilic. For instance, poly(acrylic acid) (PAAc) and poly(4-vinylpyridine) are a weak polyacid and a weak polybase, respectively. The first polymer accepts protons at low pH and releases protons at neutral and high pH. However, in the case of the weak polybase, the polymer will be protonated at high pH or positively ionized at low pH values. Controlling the pH around the hydrogel, and considering the hydrodynamic changes that occur in the polymer chain due to this pH variation, a change on the state of the material from a collapsed to an expanded state can be obtained [31]. The design of the polymer’s pendant groups is mostly related to the desirable application.

Development of hydrogels at the nanoscale has driven the group of N. Peppas [32] to recently foster systems with pH-responsive moieties for oral delivery of hydrophobic therapeutics. In this work, a set of nanoscale hydrogels, obtained from the photo-emulsion co-polymerization (ultraviolet (UV) radiation) of the methacrylic acid (MAAc) and poly(ethylene glycol monoethyl ether methacrylate) (PEGMMAA) (1:1 ratio) with the hydrophobic groups *tert*-butyl methacrylate, *n*-butyl methacrylate, *n*-butyl acrylate, and *n*-methyl methacrylate were prepared. All the hydrogels could present a transition in size induced by pH variations (4 to 7) and good physicochemical properties assessed by in vitro testing, where no considerable cytotoxicity was observed. Moreover, the same group was able to show that these nanoscale hydrogels improve the solubility and permeability of hydrophobic therapeutic doxorubicin by in vitro testing [33].

If one combines the thermal responsiveness of PNIPAAm and a pH-sensitive component as the acrylates, a pH- and thermal-responsive hydrogel is obtained. This is the basic idea behind the work of Jin et al. [34], where the authors produced smart hydrogels, based on the co-polymerization of NIPAAm, itaconic acid (IA) and methacrylate lignosulfonate. The hydrogels showed temperature-sensitive behavior, with the TvPT around the body temperature, and pH sensitivity in the range of 3.0 to 9.1. Since a wide sensitive-pH range was achieved, the authors suggest a multitude of possible applications ranging from agriculture to medicine.

### 2.3. Light-Responsive Hydrogels

The development of light-responsive materials has many advantages. For starters, light is non-invasive, and it does not require contact with the material. Moreover, light is highly accurate and has a low thermal effect [35]. Light-responsive hydrogels can be produced using different strategies, usually composed of a polymeric network and a photoreactive moiety. The photochromic molecule captures the optical signal and converts it to a chemical signal through a photoreaction (isomerization, cleavage, or dimerization) [36,37].

Azobenzenes are amongst the mostly used photo-switchable molecules. These molecules undergo *trans*-to-*cis* or *cis*-to-*trans* isomerization when irradiated with UV- and blue-light, respectively. Heat can also induce isomerization in azobenzenes due to the thermodynamic stability of the *trans* isomer [38]. Several authors have used these photoswitchable molecules to develop light-responsive hydrogels. A recent review highlights different types of light switchable azobenzene containing-macromolecules and their potential applications [39], where some examples of hydrogels were underlined.

Recently several interesting examples can be found in the literature where the authors prepared hydrogels containing azobenzene moieties. Su et al. [40] prepared starch-based hydrogel with an azo group incorporated. This hydrogel has dual-stimuli-response: photo-response due to the presence of azo groups; and pH-response due to acrylic acid and poly(vinyl alcohol) (PVA)-based macromonomers. Rastogi et al. [41] functionalized four-arm, amine-terminated PEG with azobenzoic acid. The authors used this light-responsive hydrogel to enhance the release of Alexa Fluor^®^ 750, a near infrared fluorescent dye, triggered by UV light irradiation. In both cases, the UV light irradiation induces photoisomerization of the crosslinkers from *trans*-to-*cis* isomer, which is reversible upon irradiation with visible light or dark conditions. Another research group produced a light-responsive hydrogel based on crosslinked polymers of 2-hydroxyethyl methacrylate functionalized with azobenzene groups. Upon light irradiation, the photoisomerization of azobenzene induces changes in the hydrogel polarity. In this manner, the authors were able to control the degree and rate of swelling by light irradiation [35].

Zhao et al. [42] functionalized a PEG hydrogel with *ortho*-fluoroazobenzenes as crosslinkers. Upon irradiation with blue and green light, this hydrogel exhibits reversible photo-modulation of its elasticity. This system avoids the use of UV light irradiation, which not only affects the photostability of the materials, but can also be a problem for its use in biological applications.

More recently, a new class of hydrogels have emerged. In these systems, supramolecular hydrogels are formed by self-aggregation of low molecular mass compounds to form entangled self-assembled fibrillary networks through a combination of non-covalent interactions. Since these networks are formed by weak interactions, they can be readily transformed in a fluid (sol) upon exposure to heat [1]. Mandl et al. [38] produced a supramolecular hydrogel based on PAAc-azobenzene copolymer using deoxycholate-β-cyclodextrin as a crosslinker. The authors incorporated LiYF_4_:Tm^3+^/Yb^3+^ upconverting nanoparticles in the supramolecular hydrogel, which can emit UV light upon NIR irradiation. Among the possible non-covalent interactions to form supramolecular hydrogels, the host-guest interaction is an important one. The most commonly used host molecules are cyclodextrins due to their low toxicity and better-inducing abilities [43,44]. The incorporation of an azobenzene group in its *trans* isomer into a cyclodextrin is stable in water. Upon UV light irradiation, the azobenzene group converts to *cis* isomer and repels cyclodextrin. Wang et al. [45] incorporated azobenzene into an anionic surfactant producing 6-(4-dimethylaminoazobenzene-4′-oxy) hexanoate sodium (DAH). The authors included DAH in its *trans* form in α-cyclodextrin, producing a supramolecular hydrogel by promoting hydrogen bonds between α-cyclodextrin and carboxylate in DAH and water. The presence of the dimethylamino group shifts the responsive wavelength to the region of the visible light (red), thus avoiding the use of UV light. Upon irradiation with visible light, this hydrogel showed gel-sol reversible phase transition.

Light-responsive hydrogels can also be produced through the incorporation of chlorophyllin as chromophore unit in the polymeric matrix. The incorporation of this molecule in PNIPAAm was first reported in 1990 [46]. A few years later, the same authors produced a covalently crosslinked network of PNIPAAm, sodium acrylate and chlorophyllin that can be activated using visible light, and deactivated using pH, temperature and light [47]. More recently, Xu et al. [48] produced an interpenetrated network using PAAm and PAAc with chlorophyllin incorporated. These hydrogels are not only able to respond to visible light, but also have enhanced mechanical properties caused by the incorporation of the chromophore unit.

Light-responsive hydrogels also find application in microfluidic devices. Particularly, PNIPAAm hydrogel is widely used to produce microvalves and micromixers. Using this type of material not only reduces the production cost of such devices, but also decreases the energy consumption of the device. For example, spirobenzopyran is often incorporated in PNIPAAm hydrogel to act as a molecular photo-switch triggered by light. The reader can find more useful information on this topic in a recent review [49].

### 2.4. Redox-Responsive Hydrogels

Redox-responsive hydrogels are materials able to respond to reduction and oxidation of their constituent molecular components. The activation of such systems can be chemical or electrochemical. The electrochemical activation starts ion mobility between two electrodes, thus producing a signal. More commonly, conducting polymers such as polyaniline and PPY are used to produce redox-responsive hydrogels. In these polymers, oxidation of a portion of their subunits within the polymer backbone occurs. This leads to an influx of counterions to balance the newly formed charges and a swelling response of the material [50,51].

Other polymers such as chitosan are also used to produce redox-responsive systems. Liu et al. [52] produced a synbio system able to generate two intermediates: the first redox-active chemical intermediate is an output; and a second one, a redox-capacitor that transduces the biological output response into an amplified electrical signal. The first intermediate is *p*-aminophenol and the second one is based on cathecol and chitosan. To produce the redox-capacitor, two steps are necessary: first a cathodic electrodeposition of the pH-responsive self-assembly chitosan; then an anodic electrochemical grafting of cathecol onto the deposited chitosan. The resulting system is non-conductive, but redox-active. Fu et al. [53] produced a supramolecular hydrogel composed of chitosan and zinc ions. They reported for the first time the use of this system as a disposable electrochemical sensing platform to determine hydroxyl radicals and hydrogen peroxide. In the presence of these molecules, the chitosan hydrogel disassembles, thus releasing the zinc ions. This release causes changes in the current, which is used as a signal to determine the analyte concentration.

Wojciechowski et al. [54] also produced a supramolecular redox-responsive hydrogel but with a different composition. The authors used *N*,*N*′-dibenzoyl-l-cystine as a redox-active supramolecular generator. This molecule self-assembles when triggered by pH in the presence of a reducing agent, which later enables the system disassembly. Consequently, a transient hydrogel is produced that has autonomous sol-gel-sol transitions based on the kinetic control of competing chemical reactions. The lifetimes of the transient hydrogen can be tuned either by pH or by the concentration of the reducing agent.

PNIPAAm also finds application in this type of stimuli-responsive systems. A PNIPAAm matrix incorporating covalently-bound phenylboronic acids was produced to be used as a saccharide sensing unit and a redox-active [Ru(bpy)_3_]^2+^ luminophore. The redox activity of this system can be reversed by changing the sequential stimuli (fructose and temperature), thus controlling the swelling and deswelling of the hydrogel [55].

In a different study, Zhang and coworkers [56] produced a mechanically redox-tunable hydrogel that acts by the valent transformation between ferric and ferrous ions. The hydrogel is composed of PAAc and hexadecyl methacrylate, and is reinforced by the hydrophobic association of these two molecules and by the metal ion coordination. The latter occurs between the carboxylate groups of PAAc and iron (III). The mechanical strength of the hydrogel is tuned by changing the complexation degree between PAAc and iron (III). When exposed to UV light, iron (III) is reduced to iron (II) in the presence of UV-sensitive citric acid molecules. This reaction can be reverted in the presence of oxygen, thus modulating the mechanical strength of the produced hydrogel.

### 2.5. Analyte-Responsive Hydrogels

Analyte-responsive systems are a scientific research interest since they mimic nature’s ability of molecular recognition. To design these 3D polymer networks, biomolecules capable of recognizing target molecules are incorporated in the hydrogel. These molecules can be enzymes, peptides and nuclei acids. Target analyte can be used to induce hydrogel’s assembly, disassembly, syneresis, swell and also to prompt molecular reorientation and displacement [18]. With the vast knowledge in the hydrogel field, a wide range of sensing applications have been suggested—immobilizing scaffolds for biomolecules, optical or photonic sensors, electrical or magnetic transducers or pressure-responsive, just to state a few [17,18,57].

Glucose-responsive hydrogels have been widely studied for its use in diabetes’ treatment. Moreover, the design of enzyme-responsive hydrogels is very attractive since this can act directly upon the target molecule. More recently, the production of hydrogels using a template molecule created molecularly imprinted polymers. The use of the templated molecule produces binding sites in the polymer, leading to its recognition by the desired target. Peppas’ group has recently published a review about these three classes of analyte-responsive hydrogels [7]. The reader can also find useful information about the design and production of analyte-responsive hydrogels in another recent review from the same group [18].

**Figure 4:**
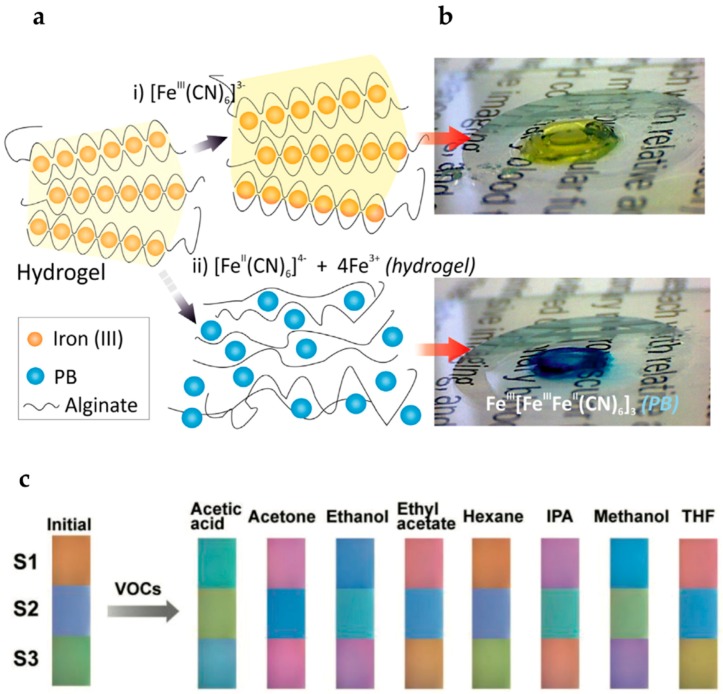
Analyte-responsive hydrogels: (**a**) schematic representation and (**b**) photographs of a bacterial metabolic activity-response hydrogel, based on an electrochromic iron(III)-complexed alginate system with antibiotic-susceptibility. In this biomaterial, the reduction of ferricyanide to ferrocyanide by the bacteria will lead to the formation of Prussian blue molecules, macroscopically observed by the strong chromatic change of the hydrogel from yellow to blue [58] (Reprinted from ref. [58]. Copyright 2018 Elsevier); (**c**) sensor array composed of poly(2-hydroxyethyl methacrylate-*co*-AAc) deposit on top of a reflective substrate with tree different thickness (234, 290 nm and 362 nm for S1, S2 and S3, respectively). When the system is expose to different volatile organic compounds one can observe a change in coloration due to the diverse swelling behavior observed for each VOC. For each VOC the system response gives rise to a specific pattern, even for similar analytes such as alcohols (methanol, ethanol, and IPA) (Reproduced from ref. [59]. Copyright 2017 John Wiley and Sons).

Pujol-Vila et al. [58] developed a very interesting biomaterial for fast antibiotic-susceptibility evaluation. This biomaterial is based on alginate complexed with iron (III) ions. Basically, the bacteria under evaluation are entrapped and pre-concentrated in the hydrogel matrix. This occurs through oxidation of iron (II) to iron (III), leading to in situ formation of the alginate hydrogel in less than two minutes in soft experimental conditions. Afterwards, the hydrogel is incubated with the antibiotic and ferricyanide is added to the biomaterial. If the bacteria are resistant to the antibiotic, they will remain alive. In this case, bacteria will reduce ferricyanide to ferrocyanide, which reacts with iron (III) ions present in the hydrogel. This reaction originates Prussian blue molecules, leading to a blue color development that is visible to the naked eye (Figure 4).

Keeping in mind the importance of nitric oxide (NO) as a signaling molecule in neurons and in the immune system, Park and co-workers [60] recently developed a therapeutic gas-responsive hydrogel. The NO-responsive hydrogel is based on the polymerization of the monomer acrylamide (AAm) as a monomer and a new synthesized NO-cleavable crosslinker the *N*,*N*′-(2-amino-1,4-phenylene) diacrylamide. This crosslinker act as a NO scavenger and when expose to NO gas the gels react with the diffuse-in NO molecules to form a benzotriazole group moiety that is then hydrolyzed, resulting in swelling of the hydrogel. The authors also showed that this system can incorporate an enzyme-triggering NO donor.

Cellulose nanocrystals (CNC) obtained from the acid hydrolysis of cellulose fibers have attracted a lot of attention from the scientific community as they are renewable, biocompatible, have low density, with high specific surface area and present interesting liquid crystalline and mechanical properties, among other [61]. Oechsle et al. [62] recently described the production of a CO_2_-responsive hydrogel based on CNC, where to a suspension of positively charge nanoparticles, an imidazole solution is added. The presence of the analyte CO_2_ triggers the gelation of the hydrogel in a pH-responsive fashion, since CO_2_ reacts with water to form carbonic acid decreasing the pH of the solution and imidazolium salt. The authors showed that if the gel was exposed to N_2,_ the 3D network collapses. The combination of CNC characteristics and its response in the form of the responsive hydrogel allowed the authors to envisage a wide range of applications, starting from switchable absorbents, and flocculants to tissue engineering.

Nature has several interesting examples where animals adapt their coloration in response to the changes in its environment. For instance, Cphalopods *Euprymna scolopes* can alter the thickness of its iridophores by stretching it, leading to a change in its iridescent coloration as a form of camouflage. In addition, structural coloration variation can be observed on the longhorn beetle *Tmesisternus isabellae*, since they change their coloration from green to red when the ambient relative humidity increases. Inspired by such examples, Qin et al. [59] developed an interferometer with adaptive coloration to be used as a vapor chemical sensor. The color adaptive sensor was produced with a simple design that consists on a single layer of hydrogel film of poly(2-hydroxyethyl methacrylate-*co*-AAc) deposit on top of a reflective substrate. The resulting colors arise from the interference of light waves reflected from the air–hydrogel interface and the hydrogel–substrate interface. The authors were able to demonstrate a color change in the presence of volatile organic compounds (VOC) as acetic acid, acetone, ethanol, ethyl acetate, hexane among others, as shown in Figure 4c. Moreover, the authors were also able to demonstrate with an on-demand patterning that these systems can be used to produce information encryption devices, triggered by humidity.

Analyte-responsive hydrogels can be key versatile diagnostic tools, as they can also create systems that are easy to use. As an example, one can see the use of a photonic biosensor array based on a interpenetrated cholesteric liquid crystal/hydrogel polymer network [63]. This system is prepared in a multi-step procedure that first give rise to a solid cholesteric liquid crystal film in which a hydrogel interpenetrated array is then built using a UV photomask template. In this set, the enzyme is then immobilized, giving rise to the biosensor array. The complexity of the preparation set-up is antagonistic to the oversimplified nature of its use since the biosensor uses a change in color system to detect the target analyte, as the authors demonstrated for the analyte urea.

### 2.6. Hydrogels for 3D Printing

If the interesting characteristics of stimuli-responsive hydrogels presented above are considered, one can envisage its use in 3D printing systems or bioprinting or biofabrication, in which one can accurately develop structures, as scaffolds for tissue engineering, or printing cells or even organs [64,65]. The most commonly used hydrogels inks comprise calcium of alginate, or include colloidal suspensions and polyelectrolyte gels [66]. One should note that a hydrogel can be printable if it demonstrates shear-thinning behavior under pressure, this is, if it acts as a non-Newtonian fluid (its viscosity is dependent on the applied shear) allowing, for instance, normal thick liquids or gels to flow freely. However, the knowledge of printing parameters is of extremely importance in order to be able to precisely control the hydrogel depositions, especially if one considered the printing of 3D structures that mimic human organs. Keeping this in mind, He et al. [65] were able to establish relationships between printing parameters, such as air pressure, feed-rate, or even printing distance and printing quality of the expected structures.

Tan et al. [64] recently presented an innovative set-up to produce 3D printing structures that mimic the mechanical properties of the softest tissues found in the human body (for instance brain and lung tissues). In this work, a liquid ink of PVA:Phytagel (agar substitute gelling agent) is cryogenic printed, with an extrusion-based method, on an isopropanol bath with dry ice and solid CO_2_. In this set-up, the authors use the liquid-to-solid phase change to obtain 3D hydrogel structures, by rapidly cooling the ink below its freezing point. When coated with collagen type I, the hydrogels show good cell attachment and viability. The use of cryogenics to obtained printed super soft hydrogels led the authors to postulate several possible applications, which range from soft tissue for surgical training and simulations to mechanobiology and tissue engineering.

Multi-stimuli-responsive hydrogels inks for direct-write 3D printing were developed by Karis and co-workers [66]. In this essay, the authors presented to the reader cleverly designed triblock copolymer hydrogels based on living anionic ring-opening polymerization of glycidyl ethers, as allyl glycidyl ether (AGE), with isopropyl glycidyl ether (iPGE) from a PEG macroinitiator. In doing so, the authors could combine the thermo-responsiveness of PiPGE-*b*-PEG-*b*-PiPGE with the cross-linkable functionality of PAGE-*b*-PEG-*b*-PAGE and form a series of PiPGE-*stat*-PAGE-*b*-PEG-*b*-PiPGE-*stat*-PAGE macromolecules. These polymers can self-assemble in water and give rise to hydrogels that respond to temperature, pressure, and UV light. These features help produce a robust free-standing 3D printed object.

Yang et al. [67] recently presented a functional hybrid ink for 3D printing. The authors, using PNIPAAm hydrogel within a water-rich silica-alumina (SiO_2_/Al_2_O_3_)-based gel matrix, were able to obtain a hydrogel that can take up to 70 wt. % in water, respond to temperature and electric stimuli, and be printed using a commercial 3D printer. The hybrid hydrogel is transparent below PNIPAAm’s LCST and opaque above, due to the gels’ dehydration. Using this feature, the authors printed this hybrid system into a flexible electro-thermochromic device. This device allows the authors to display the electric/temperature tunable transmittance of the systems, with lower response times when compared to other reported systems, as well as its bending properties (the system sustain bending angles of 180° without feature failure).

## 3. Multi-Stimuli-Responsive Hybrid Microgels

Polymeric microgels are in an intermediate state between the branched polymers and macroscopic networks, with a molecular weight (Mw) comparable to that of a linear high Mw polymer, however with a structure resembling that of a macroscopic network. This indicates that in an appropriate solvent, they swell instead of dissolving, forming a colloidal dispersion [68]. Depending on the composition, microgels can be sensitive to external stimuli, a feature that has generated much interest because of its versatility for potential application. Pelton and Chibante [69] synthesize for the first time thermoresponsive PNIPAAm microgel particles that shrinked upon heating due to the intrinsic LCST that NIPAAm monomer possesses. This kind of thermoresponsive microgels is defined as negative thermorsensitive. However, there are also positive thermosensitive microgels, albeit less studied. An example of the latter is the system formed by the poly(acrylamide-*co*-acrylic acid) (PAAm-*co*-AAc) copolymer, which swells upon heating due to the breakage of hydrogen bond interactions (the so-called zipper effect) [70].

PNIPAAm is undoubtedly the most studied thermo-responsive microgel [71,72,73,74,75,76,77]. The NIPAAm monomer is often used to produce microgels using precipitation polymerization. In this technique, the initially produced polymers are insoluble in the used solvent (usually water) at the reaction temperature (above LCST). The initial nucleation period determines the number of particles, and is followed by the growth period, in which the freshly polymerized polymer adds onto the preformed nuclei. In the presence of a suitable crosslinker, the polymer chains remain in the network and only when the temperature decreases (below LCST), the PNIPAAm microgels swell in water [78]. More information related to microgel synthesis can be found in a recently published book [79].

Although plain PNIPAAm microgels are often used in several applications, the incorporation of additional functional monomers gives raise to more complex and interesting microgels. The addition of co-monomers usually affects the swelling behavior of the microgel. PNIPAAm, in particular, is often functionalized with organic acids (e.g., AAc [80,81], MAAc [82,83]), which introduce changes in the polymer network, thus influencing its swelling behavior [76]. The use of acrylamide-based monomers is also advantageous due to the presence of at least one vinyl group that participates in the polymerization reaction and/or crosslinking process. Furthermore, non-acrylamide based monomers such as *N*-vinylcaprolactam originate pH-responsive microgels [84].

Core-shell microgels of PNIPAAm have also been explored due to the possibility of combining the different characteristics of both the core and the shell. For example, core-shell microgels composed of a polystyrene core and a PNIPAAm shell [85] have been studied for catalysis applications [86] and to produce composite membranes with tunable permeability [87]. In a different approach, Brugnoni et al. [88] produced core-shell-shell microgels using silica nanoparticles as the core, poly(*N*-isopropylmethacylamide) (PNIPMAAm) as the inner shell, and PNIPAAm as the outer shell. In addition, by dissolution of the silica core, the authors produced hollow microgels, which enable the production of a series of microgels with diverse thermal response from the initial composition. Zha et al. [89] produced thermoresponsive microcontainers for delivery applications, also using silica nanoparticles as templates with a PNIPAAm shell.

Using these few examples, we tried to give the reader a glance of the multiple possiblities of homo- and co-polymeric microgels constructs. It is not our intention to cover every type of microgel and its application. Several reviews have been published recently covering different aspects of microgel design and synthesis, and their applications [75,76,78,84,85,90,91,92,93,94]. As indicated in the introduction, much effort has been devoted to the obtention of advanced materials able to responde to several external stimuli. In this regard, the strategy followed consisted of the formation of hybrid systems. By definition, a hybrid material results from a combination at the nanometric and molecular level of an inorganic and an organic component, allowing the preparation of new multifunctional materials [95]. The properties of hybrid materials emerge not only from the individual properties of each component, but also from synergetic effects from the interaction of the components. In the case of hybrid polymeric gels, the inorganic component is generally based on nanoparticles not only due to their quantum size effects but also to take advantage of their high surface to volume ratio [96]. Therefore, in this section, we intend to describe the most recent stimuli-responsive hybrid polymer microgels, focusing on the type of nanoparticles embedded in the polymeric matrix.

### 3.1. Metallic Nanoparticles

#### 3.1.1. Gold Nanoparticles

Gold nanoparticles (AuNPs) have been used since 1857 when Faraday discovered the different properties of colloidal gold and bulk gold [97]. Nowadays, AuNPs are used in analytical chemistry [98], electronics [99], as well as biology and nanomedicine due to their characteristic properties such as tunable optical properties, biocompatibility, high surface area, and can undergo surface modification [100].

**Figure 5:**
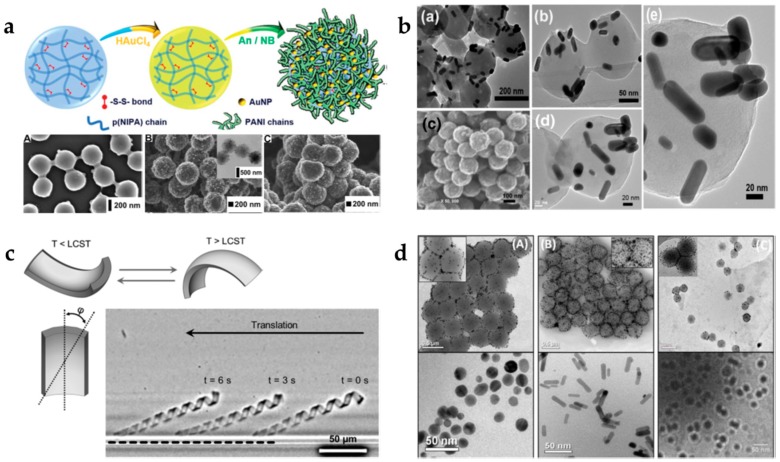
(**a**) Scheme for the preparation of PNIPAAm microgels, polyaniline (PANI) fibers and AuNPs microcomposite and the respective scanning electron microscopy images (Reproduced from ref. [106]. Copyright 2016 The Royal Society of Chemistry); (**b**) Transmission electron microscopy images of hybrid microgel particles composed of PNIPAAm microgels and AuNRs. (a,b,d,e) The figure show spherical microgels with a rough surface with Au NRs attached; (c) Field emission scanning electron microscopy image of hybrid microgels (Reproduced from ref. [107]. Copyright 2013 John Wiley and Sons); (**c**) Schematic illustration of the helix direction controlled by the principle curvatures 1/R1, 1/R2 and the mismatch of ϕ toward the length axis of the ribbon. Directing the rotational motion to a linear translocation when the oscillating helix is confined close to a flat wall that impedes the rotation around the axis normal to the helix direction. The helix contour length is 160 μm, the dashed line indicates the wall position (Reproduced from ref. [108] Copyright 2015 John Wiley and Sons); (**d**) Transmission electron microscopy images of (A) PNIPAAm-Au nanospheres (B) PNIPAAm-AuNR and (C) PNIPAAm-Ag nanospheres [109] (Reprinted from ref. [109]. Copyright 2017 Elsevier).

The most common organic component of a hybrid polymer gel with gold nanoparticles is PNIPAAm. Several research groups have recently used AuNPs incorporated into PNIPAAm microgels for catalytic applications. Shi et al. [101] used AuNPs with 3.8 nm in diameter homogeneously incorporated into thiol-functionalized PNIPAAm microgels by in situ reduction of HAuCl_4_ aqueous solution based in Au-thiol chemistry. By using this methodology, the authors were able to obtain control over the distribution of AuNPs within the microgel. In another case [102], the authors used AuNPs incorporated into a PNIPAAm-based copolymer (poly(NIPAAm-*co*-allylamine)), which responds to both temperature and pH stimuli. With this hybrid system, the authors were not only able to modulate the catalytic activity of AuNPs, but also allow label-free in situ localized surface plasmon resonance (LSPR) monitoring of the catalyzed chemical reaction. The modulation is performed by varying the solution temperature, while the monitoring is made through stimuli-responsive volume phase transitions of microgels, thus changing the immediate physicochemical microenvironment of AuNPs, resulting in an alternation of the LSPR. Similarly, Rehman and coworkers [103] crosslinked NIPAAm and *N*,*N′*–dimethylaminoethylmetacrylate (DMAEMA) in water to obtain microgels responsive to temperature and pH. The poly(NIPAAm-*co*-DMAEMA) microgels were used as nanoreactor for the synthesis of AuNPs. These microgels demonstrated catalytic properties for the reduction of toxic 4-nitrophenol (4-NP). Moreover, the catalystic activity can be tuned using the microgels’ thermobehaviour. Chen and his team used photoluminescent gold nanodots with 1.8 nm in diameter incorporated into PNIPAAm microgels for the detection of mercury ions (Hg^2+^). This detection is based in the formation of Au-Hg amalgam and hybrid microgel aggregates in the presence of Hg^2+^, leading to decreased photoluminescence of the hybrid microgels. By using these hybrid microgels, the authors were able to sensitively and selectively detect Hg^2+^ ions in environmental and biological samples [104]. Tang et al., used the same principle to produce hybrid microgels for the detection of Hg^2+^ by using poly(NIPAAm-*co*-2-(dimethylamino)ethylmethacrylate) microgels [105]. Mackiewicz and coworkers [106] produced a new multifunctional microcomposite by combining three components: PNIPAAm microgels, polyaniline (PANI) fibers and AuNPs (Figure 5a). PANI is one of the most common and interesting conducting polymers. The combination of PANI nanofibers with AuNPs led to a substantial increase in conductivity and electroactivity of the microcomposite. Thus, the microcomposite showed much stronger electrocatalystic properties for ethanol oxidation reaction in alkaline medium compared to bare gold electrodes.

Other authors used gold nanorods (AuNR) located at the surface of the microgel instead of spherical nanoparticles incorporated into PNIPAAm microgels (Figure 5b). In both cases, the stimuli-responsiveness of the microgel (temperature and pH), which causes a significant change in the aspect ratio (length/width) of the hybrid microgel, created an important shift in the UV-VIS absorption intensity and a shift in the longitudinal surface plasmon bands of the gold nanorods [107,110].

Mourran et al. [108] used the particle replication in non-wetting templates (PRINT) technique to produce PNIPAAm microgels with gold nanorods to act as soft microbots. Temperature-responsive thin PNIPAAm microgel bodies can undergo bending and torsional motions upon sweeling and unsweeling (Figure 5c). The addition of AuNRs promote very fast temperature jumps of more than 20 °C within less than miliseconds by their photothermal heating. The authors demonstrated that for the proposelfully shaped microgels, the kinematics of a cyclic body shape variation can be tuned to differentiate between the forward and the backward motion.

A common strategy for the incorporation of gold NPs into polymeric microgels is through the in situ reduction method. Liz-Marzán and coworkers [111,112,113] have developed a method to encapsulate AuNPs within PNIPAAm microgels. The authors found that thermo-responsiveness behavior and crosslinking density of the microgel allow efficient regulation of the catalytic activity of the AuNP. Moreover, this composite can be reusable for several cycles with its catalytic property almost perfectly reproducible [114]. Shi et al., used P(NIPAAm-*co*-MAAc) to obtain thermo-, pH-, and light-responsive hybrid microgels. The authors produced the polymeric microgels, then thiol-functionalized them and obtained the hybrid microgels by in situ reduction of the gold precursor in the presence of the microgels based on Au-thiol chemistry, achieving a fluff-like structure due to the numerous AuNPs distributed in the interior of the microgel [115]. In a different study, Agrawal and her team used *N*-vinylcaprolactam/acetoacetoxyethyl methacrylate/acrylic acid-based microgels (P(VCL-AAEM-AAc)) as a polymer template for in situ reduction of the gold precursor. The colloidal gold nanostructure is selectively formed in the core of the microgel and the hybrid structure is used as a noble metal catalyst [116]. PVCL has a similar thermosensitivity to PNIPAAm, i.e., it exhibits LCST in aqueous media and goes through coil-to-globule transitions upon heating. However, PVCL is more biocompatible than PNIPAAm since upon hydrolysis, the resultant monomers are less cytotoxic. Consequently, some authors have used PVCL for the construction of hybrid microgels incorporating AuNPs for biomedical purposes [117,118].

In a different study, thermoresponsive PNIPAAm-metal hybrids were produced using PNIPAAm microgels as a microreactor. Metal NPs of different shapes were embbebed in the microgel: Au nanorods, Au nanospheres, and silver (Ag) nanospheres (Figure 5d). These core-shell microgels were used to catalytically convert 4-NP to 4-aminophenol (4-AP). The authors conclude PNIPAAm-AuNPs and AuNRs have better catalytic activity, as well as recyclability compared to that of PNIPAAm-AgNPs [109]. Similarly, Shah et al. [119] produced multiresponsive tercopolymer microgels (poly(NIPAAm-*co*-MAA-*co*-2-hydroxymethylmethacrylate) with homogeneously dispersed Ag and AuNPs. These microgels exhibit sensitivity towards temperature and pH. In addition, terpolymer microgels are efficient catalystics for the reduction of 4-NP, congo red and methylene blue in waste waters.

#### 3.1.2. Silver Nanoparticles

Silver nanoparticles (AgNPs) being one of the noble metals, have unique optical, electrical and surface properties. These nanoparticles are commonly used as sensor, optical switches, antimicrobial agents, and catalysts [120,121].

Similarly to AuNPs, silver-based hybrid microgels are generally composed of thermosensitive polymers, most commonly PNIPAAm. In addition, a copolymer is often used to obtain a dual-stimuli-responsive microgel, for example, a temperature- and pH-sensitive microgel. Liu et al. [122] prepared PNIPAAm microgels with AgNPs by in situ reduction of Ag^+^ coordinated into monodisperse PNIPAAm microgels, in which the catalytic activity can be tuned with four stages of change versus temperature. With the same purpose, Tang et al. [123] used P(NIPAAm-*co*-DMA) for in situ reduction of Ag^+^ ions pre-dispersed into the microgels. These hybrid microgels possess thermo- and pH-responsiveness, as well as catalytic activity. In a different study, Khan et al. [120] used poly(NIPAAm-*co*-allylacetic acid) (P(NIPAAm-*co*-AAAc)) with AgNPs incorporated into the microgel by in situ reduction to be used as catalysts for reduction of nitrobenzene into aniline, exploring various conditions of temperature, catalyst dose and concentration of the reagents. The authors found that the catalytic activity of the hybrid microgels can be tuned by changing the temperature of the medium.

As previously stated, AgNPs can also be used in sensor devices. For example for glucose sensing, Khan et al. [124] performed in situ reduction of silver nitrate in the presence of glucose using P(NIPAAm-*co*-AAc) microgels as a template, while Zhou et al. [125] used AgNPs immobilized in a glucose-imprinted boronate derivated gel network of poly(NIPAAm-*co*-acrylamide-*co*-4-vinylphenylboronic acid) (P(NIPAAm-*co*-AAm-*co*-VPBA)) as a glucose sensing element. Another research group used AgNPs incorporated into poly(3-acrylamidophenylboronic acid-*co*-2-(dimethylamino)ethyl acrylate) microgels as glucose sensors. The glucose-sensing performance of the hybrid system was improved by tailoring the phase behavior from monotonous shrinking upon adding glucose, as well as the following change in the signaling manner from “turn-off” to “turn-on” [126].

PNIPAAm-based hybrid microgels are also used as sensors for other molecules, for example H_2_O_2_ colorimetric sensing. Han and coworkers developed P(NIPAAm-*co*-AAc) microgels with AgNPs incorporated that went through autocatalytic oxidization in the presence of H_2_O_2_. The authors found that the absorbance at 400 nm was linearly dependent of H_2_O_2_ concentration and that was selective [127].

Kumacheva’s group produced AgNPs in pH-responsive microgels of P(NIPAAm-AAc-2-hydroxyethyl acrylate) and found that the AgNPs produced in situ, i.e., through photoreduction of Ag^+^ ions were fluorescent, while the AgNPs produced from conventional method do not exhibit this property [128]. Similarly, other research groups have used this property of in situ produced AgNPs to obtain hybrid microgels for fluorescent detection [129,130].

Finally, AgNPs-based hybrid microgels are also used for plasmonic sensing due to the coupling between the oscillating electromagnetic fields on the AgNPs surface originating from plasmon resonance, which provides significant enhancement of the local electromagnetic fields. As such, these systems can be used as substrates for surface-enhancement raman scattering sensing. For this application, the plasmonic properties and surface electromagnetic field enhacement effects of the AgNPs present in the hybrid microgels are tuned by temperature and pH, for example, thus resulting as mobile plasmonic or SERS microsensors to detect environmental temperature or pH variation [121,131].

### 3.2. Magnetic Nanoparticles

Magnetic nanoparticles such as superparamagnetic iron oxide nanoparticles (Fe_3_O_4_, SPIONs) are widely used for biomedical applications due to their biocompatibility and interesting magnetic properties. The main biomedical applications of this type of nanoparticles are magnetic hyperthermia agents or contrast agents for magnetic resonance image [132,133,134,135,136,137]. The combination of polymeric microgels with Fe_3_O_4_ NPs allows the construction of a composite system with the properties of the polymer with magnetic-responsive properties. For example, several authors incorporated Fe_3_O_4_ NPs into PNIPAAm microgels, obtaining a thermo- and magnetic-responsive system. In some cases, the iron oxide NPs act as a crosslinker for PNIPAAm microgels, assuring a higher dispersibility of the NPs in the polymeric network [138,139]. In addition, the heating ability of the Fe_3_O_4_ NPs may provide an in vitro burst release of chemotherapeutic drugs [140,141].

Boularas et al. [142,143] were able to encapsulate a high content of magnetic NPs (up to 33 wt. %) into poly(di(ethylene glycol)) methyl ether methacrylate-*co*-oligo(ethylene glycol) methyl ether methacrylate-*co*-methacrylic acid) microgels, obtaining thermo-, pH-, and magnetic-responsive microgels. For the same purpose, other authors used different polymers for the production of the microgels, such as P(VLC-*co*-IA) based microgels [144], poly(2-(2-methoxyethoxy) ethyl methacrylate-*co*-oligo(ethylene glycol) methacrylate-*co*-AAc) incorporated with attapulgite/Fe_3_O_4_ NPs [145,146], among others.

Zhou et al. [147] developed a facile one-pot synthesis method of Fe_3_O_4_-PVA microbeads for drug delivery applications. The beads are prepared by dropwise addition of mixed aqueous solution of iron salts and PVA solution into alkaline solution. To add a thermo-responsive functionality to the beads, the authors added PNIPAAm to the mixture, creating magnetic gel beads with high drug loading capacity that have magnetic and temperature-responsiveness.

Other authors produced poly(vinyl phosphonic acid) nanogels with silica-coated Fe_3_O_4_ NPs. They also produced porous nanogels by using triethoxyvinylsilane as co-monomer during polymerization, followed by removing the silica by hydrofluoric acid treatment after polymerization. These nanogels were used for two main applications: (i) drug delivery systems of zuclopenthixol and phenazopyridine hydrocholride as models drugs; and (ii) absorbents for removal of organic contaminants such as 4-NP, 1,1′-dimethyl-4,4′-bipyridinum dichloride (Paraquat), methylene blue, and rhodamine 6 G from aqueous media [148].

The combination of polymer gels with Fe_3_O_4_ NPs can also be used for catalytic applications. Liu et al. [149] prepared Fe_3_O_4_-P(MBAAm-*co*-MAAc) microspheres that contained carboxyl groups for application as magnetic catalyst support to load a series of metallic nanoparticles such as Ag, Pt, and Au. The Fe_3_O_4_ NPs loaded into the microgels were able to maintain their superparamagnetic behavior, together with their high saturation magnetization, providing a convenient way for separating metallic nanoparticles from a solution. In a different study, Nabid et al. [150] incorporated Fe_3_O_4_ NPs into P2VP microgels where the magnetic NPs acted as crosslinkers. These hybrid microgels are able to trap metal ions such as Pd^2+^ via complex formation of P2VP with metal ions, and the reduction of the Palladium ions by sodium borohydride led to the formation of PdNPs@P2VP-Fe_3_O_4_ hybrid microgels. These systems may be employed as nanocatalysts toward oxidation reaction of alcohols, which can be modulated by the volume phase transitions of the microgel.

### 3.3. Carbon-Based Materials

Carbon nanotubes (CNTs) are often used as reinforcement agents to create porous hydrogels with tunable mechanical properties. The incorporation of CNTs into hydrogels increases not only later elastic modulus, but also the thermal stability and electric conductivity of the resulting composites [151,152].

**Figure 6:**
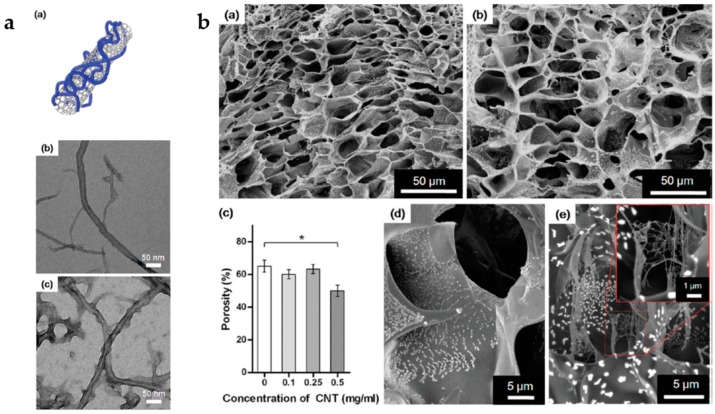
(**a**) CNT-gelatin methacrylate (GelMA) hybrids: (a) Schematic diagram of a GelMA-coated CNT and the respective high resolution transmission electron microscopy images of bare CNTs (b) and GelMA coated CNTs (c); (**b**) Scanning electron images of cross sections of GelMA (a) and CNT GelMA (b) hydrogels with 0.5 mg/mL CNTs; (c) CNT-GelMA hydrogels porosity with different concentrations of CNTs (* *p* < 0.05); Magnified images show parts of a cross section of (d) GelMA and (e) CNT GelMA hydrogel with nanofiber junctions inside a porous structure. (Reprinted with permission from [152]. Copyright 2012 American Chemical Society).

Shin et al. [152] used reinforced CNT-gelatin methacrylate hybrid as a biocompatible, cell-responsive hydrogel platform to create cell-laden three-dimensional constructs (Figure 6). These hybrids are photopatternable, which allow easy fabrication of microscale structures without harsh processes. In a different study, Spizzirri and coworkers produced gelatin/CNT hybrid hydrogels as effective diclofenac sodium salt carriers. The drug is released in response to an electric stimulation [151]. Cui and coworkers [153] produced conductive gel composites using only colloidal particles as building blocks. These composites are composed of mixed dispersions of vinyl-functionalized pH-responsive microgel particles and multi-walled CNTs. The microgel particles act as dispersant and macrocrosslinkers, while CNTs provide electrical conductivity and modulus enhancement. These gel composites showed promising properties to be used as injectable gels for application in soft tissue repair and electronic skin.

Zhou et al. [154] used the technique previously described for AgNPs [125], but instead of silver, the authors went further and added carbon dots as the inorganic part to the polymeric matrix (P(NIPAAm-AAm-VPBA)) to produce hybrid microgels for optical sensing of glucose. This new sensor is able to continuously monitor glucose level change in physiological conditions.

Among carbon-based materials, graphene oxide (GO) obtained from chemical modification of graphite is considered a molecular two-dimensional (2D) platform with a high aspect-ratio, also being water-dispersible. This material has several advantages such as high mechanical strength, pH sensitivity, photosensitivity, and low toxicity. In fact, GO shows higher photothermal sensitivity than CNTs, and is capable of efficiently converting optical energy into thermal energy [155,156].

Lu et al. [157] incorporated chemically-reduced GO into PNIPAAm microgel for the preparation of photo- and thermoresponsive composite microgels for drug delivery applications. Wang et al. [156] used the same idea but the chemically reduced GO was incorporated into a thermosensitive nanogel composed of acrylated chitosan, NIPAAm, and PEG diacrylate incorporated with doxorubicin. The composite nanogel exhibited a near infrared (NIR)-induced thermal effect with a high doxorubicin loading capacity. Moreover, doxorubicin release appears to be faster when the composite nanogel is irradiated with NIR light. Graphene oxide composite microgels also found applications as selective glucose-responsive systems for photoluminescent detection of glucose in blood serum [158] or as biocompatible injectable composite gels to repair load supporting soft tissue [155], among others.

Finally, Girard et al. [159] synthetized thermoresponsive nanodiamonds hybrid microgels using PNIPAAm as the organic component. The results showed that the microgels kept the thermosensitivity of PNIPAAm, while achieving a 2D organized array of nanodiamonds, allowing the cost-limited nanoscaled nanodiamonds organization on a substrate with great flexibility for technological or optical applications.

### 3.4. Other Inorganic Materials

Among the different inorganic materials used to produce hybrid polymeric microgels, silica nanoparticles are of great interest for the production of smart self-catalyzing systems. Agrawal et al. [160] produced composite microgel particles containing silica NPs by simultaneously converting PEG-poly(ethoxysiloxane) and deposite silica in the microgels. In this case, the incorporation of silica NPs increased the rigidity of the microgels and reduced their thermosensitivity. Similarly to other inorganic materials, PNIPAAm is the most commonly used polymer for the production of silica-based hybrid microgel systems [77,161], for example for drug delivery applications [162]. Dechézelles et al. [163] synthesized thermosensitive raspberry-like hybrid microgels consisting of a PNIPAAm core decorated with in situ formed silica particles. These hybrid microgels provide a convenient basis for additional surface modifications through silane coupling agents.

Nanoclays such as Laponite have been introduced into microgel systems for the development of responsive scavenger systems due to the cation-exchange capabilities of clays, thus allowing charging of the microgel with (cationic) metal precursors. For this purpose, Contin et al. [164] developed a ternary system composed of Laponite NPs incorporated into poly(VCL-*co*-acetoacetoxyethyl methacrylate) PVCL/AAEM microgels loaded with different metal NPs: Pd^2+^, Au^+^, Pt^+^. The results showed that some of the synthesized microgels are active catalysts for the Suzuki reaction of aryl iodides in a water-rich medium. In a different application, Du and coworkers [165] produced PAAm/hectorite (Laponite^®^ XLS) double-network hydrogels containing neutral PAAm microgels, obtaining a tough and highly stretchable microgel-reinforced hydrogel with superb mechanical strength. In this case, Laponite acts as a physical crosslinker of the hydrogels.

**Figure 7:**
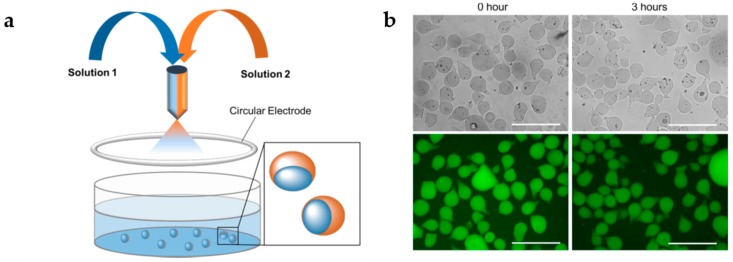
Fabrication of the alginate-based multi-compartment microgel beads with CdTe QDs incorporated: (**a**) Schematic diagram showing fabrication of multi-compartment microgel beads using the microfluidic electrospray technology; (**b**) Representative phase-contrast and fluorescence images of the QD-loaded microgel beads captured at different time points. The scale bar is 500 μm (Reprinted with permission from [169]. Copyright 2016 American Chemical Society.)

Another class of interesting inorganic materials are quantum dots (QDs), a semiconductor nanostructure that can be used for bioimaging and labeling probes. Their combination with polymer microgels extends the range of application for optical sensing, imaging diagnostics, and controlled drug release [166]. For example, Wu et al. [166] produced hybrid nanogels based on in situ immobilization of CdSe QDs in CS-PMAAc networks. These hybrid nanogels have excellent colloidal and structural stability and are pH-responsive. Moreover, the results suggest that covalently crosslinked nanogels are more suitable for optical pH-sensing and bioimaging with low-cytotoxicity, compared to physically associated hybrid nanogels. In a different study, Gui and coworkers [167] produced CdTe/ZnS QDs incorporated into P(NIPAAm-*co*-AAc) microgels by facile electrostatic attractions at room temperature. These hybrid microgels are thermosensitive, with almost fully reversible photoluminescence between 25 °C and 45 °C, and bright fluorescence imaging. Similarly, Cai et al. [168] incorporate cysteamine-capped CdTe QDs in P(NIPAAm-*co*-AAc) microgels. The photoluminescence intensity and color of the hybrid microgels can be tuned by adjusting the QDs content. In a different approach, Lai et al. [169] used microfluidic electrospray technology to produce alginate-based multicompartment microgel beads with CdTe QDs incorporated (Figure 7). These microgel beads can effectively separate incompatible drugs during co-delivery and significantly prolong the time of fluorescence emission from QDs.

Cui et al. [170] produced hybrid polymeric microgels using a different type of polymer. The authors used a imidazolium-based poly(ionic liquid) to prepare monodisperse microgels by means of microfluidics. They found that the imidazolium units in the microgel network may be exploited as reactive sites for functionalization by a simple counteranion-exchange or conversion reaction. As a proof-of-concept, the authors produced three types of isotropic functional particles, including metal-polymer hybrid particles, conductive composite particles, and catalytic particles, from poly(ionic liquid) microgels, demonstrating the versatility of these systems.

### 3.5. Multifunctional Hybrid Fibrillary Gels

In a different approach, multi-stimuli-responsive systems can be produced using stimuli-responsive microgels as active sites incorporated into polymeric fibers. Using colloidal electrospinning, it is possible to design multifunctional, highly porous, and biocompatible membranes suitable for several applications.

Electrospinning is a simple, fast and easy to scale-up technique to produce functional materials based on polymeric fibers with diameters ranging from 2 nm to several micrometers. Electrospun micro/nanofibers have a high surface-to-volume ratio, tunable porosity, and can be produced from natural or synthetic polymers [171,172,173,174]. This technique involves the application of an electrostatic force to generate a polymer jet towards a collector electrode. Despite the simplistic setup, the theory behind this technique is more complex and occurs in three stages: initiation of the jet, elongation, and fiber formation [172,175].

A variation of traditional electrospinning technique is colloidal electrospinning, where the polymeric solution is replaced by a colloidal dispersion. The presence of particles in the spinning solution allows the formation of core-shell fibers from a single nozzle, offering a simpler setup than that used in coaxial electrospinning, which needs two or more needle-tips [176,177]. The presence of small amounts of a fiber forming polymer, acting as a template, is usually required to produce composite fibers from colloidal dispersions containing either inorganic or organic (polymeric) particles [176].

**Figure 8:**
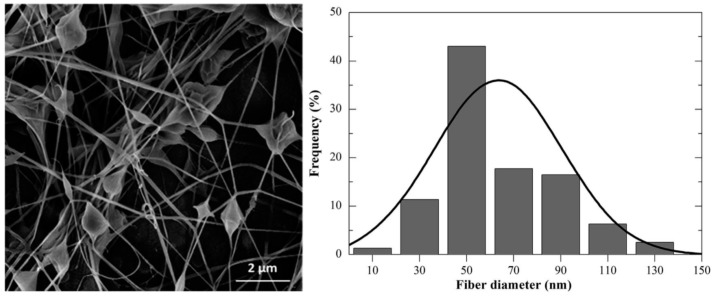
Scanning electron microscopy image optimized electrospun fibers containing PNIPAAm microgels (on the **right**) and fiber diameter distribution with a mean fiber diameter of 63 ± 25 nm (on the **left**) (Reprinted from ref. [71]).

Colloidal electrospinning allows the formation of materials with hierarchical levels of nanostructures, given by the particles and fiber morphology. Moreover, the immobilization of particles in nanofibers allows easy handling and separation from a reaction medium, in contrast to particle suspensions. More commonly, inorganic particles are used in colloidal electrospinning. These particles have higher electronic density than the polymer templates, and can be easily localized in the fibers by electron microscopy [178]. However, the confinement of stimulus-sensitive microgels in fibers by means of colloidal electrospinning could be an interesting approach towards the production of multifunctional fibers with fast thermoresponsive behavior and super-hydrophobic tunable surfaces. This may be used in drug delivery systems, bio-sensing, chemical separation, catalysis, and optics [179]. Few studies reported the confinement of crosslinked PNIPAAm microgels inside nanofibers. For instance, Nieves et al., produced composite electrospun fibers of PNIPAAm microgels (up to 40% of microgels per-centage mass) using poly(vinyl pirrolidone) (PVP) (which is a hydrogel itself) as fiber template with a mean fiber diameter of 0.9 μm [179]. Tunable surfaces of electrospun non-woven mats with PNIPAAm microgels/poly(l-lactic acid) fibers, in which the production of fibers with a mean fiber diameter of 284 nm connected to bead sizes of 3.4 μm with a spindle-like structure, was reported by Gu et al. [180]. Recently, PNIPAAm based microgels were confined in electrospun fibers using colloidal electrospinning technique. In the first case, PNIPAAm and PNIPAAm-CS crosslinked microgel dispersions were prepared by means of surfactant-free emulsion polymerization using *N*,*N*′-methylene bisacrylamide as a crosslinking agent. These thermosensitive microgels were successfully confined in poly(ethylene oxide) (PEO) fibers via colloidal electrospinning, resulting in beads randomly distributed over the fibers with a typical “bead-on-a-string” morphology. Furthermore, by performing a statistical analysis, the relationship of the processing variable over the fiber diameter was evaluated using the response surface methodology. Using the set of optimized parameters, PEO fibers with an average diameter of 63 nm containing PNIPAAm microgels, were produced (Figure 8) [71].

In a later study, PVP was used as a fiber template to produce multifunctional fibrillary gels using PNIPAAm-based microgels as active sites. PVP has the advantages of being biocompatible and water-soluble, thus avoiding the use of organic solvents. In addition, this polymer can be easily crosslinked using UV radiation, therefore avoiding its immediate dissolution under physiological conditions. Composite fibers produced by colloidal electrospinning and crosslinked for 30 min were able to swell about 30 times their weight after 1 h in aqueous medium [73].

These works are a step forward in the design and development of new and improved smart systems that can be tailored for the desired application —biomedical or industrial. One example is to use different types of microgels to create a multiresponsive membrane. Another approach will be to use hybrid microgels instead, thus taking advantage of the dual-responsivess of these type of systems, combined with the fiber’s properties.

## 4. New Generation of Functional Stimuli-Responsive Polymer Hydrogels: Self-Shape (Shape-Memory) and Self-Healable Systems

### 4.1. Stimuli-Responsive Shape Memory Hydrogels

Materials that show shape memory effect are able to deform on a new temporary shape and store in this state with a further controlled recovery of its original shape when an external stimuli (typically temperature) is applied above a critical value [181,182]. For instance, Shape Memory Polymer (SMP) systems have facilitated the development of devices for biomedical applications such as stents, dialysis needles, drug carriers, drug delivery systems, etc. [183,184,185]. However, the problem of SMP systems is the difficulty to be applied for a biological media in a living system, mainly due to the high temperatures that are needed to deform the polymers. Such temperatures could damage the surrounding tissues and even the drugs’ activity that are carried out and further delivered. In order to solve such problems, in recent years, shape memory hydrogels that are predominantly water, have postulated as alternative candidates as potential systems for biomedical applications due to the use of water as the driving force for the desired shape memory effect [186,187,188].

The development of shape memory hydrogels faces some difficulties in terms of glass transition temperature, which is usually the phase transition used for the shape stabilization and shape recuperation in SMP. Basically, the presence of a large content of water molecules carries a loss of efficiency in hydrogels. The main strategy to overcome this problem is to promote the formation of new crosslinking in the different deformation states of the hydrogel. Such new links allow the storage of shape. Breaking the new crosslinking points will end up in recovery of the initial shape.

In 1995, Osada et al. [189] reported for the first time the phenomenon of “shape memory” effect in polymer hydrogels. They showed how the temperature-dependent poly(AAc-*co*-*n*-stearyl acrylate) swells with temperature. At temperatures below 25 °C, the gel behaves as a hard system, whereas above 50 °C it, becomes soft and stretchable (up to 1.5 times its initial length). Authors suggested that the shape memory effect came from the temperature-induced order-disorder transition associated with alkyls side chains and stearyl acrylate unit interactions. In addition, authors highlighted that the shape “remembered” by the gel was dependent on the container where it was first formed.

Despite this pioneer study, hydrogels that can undergo shape transitions (temporary-to-permanent) while containing large amount of water solvent are still scarce. Here, we report some of the most recent and relevant achievements (last five years) in the subject.

Among the polymer matrices constituting shape memory hydrogels, three are more commonly used: PAAm, PVA and PEG. For instance, Zhang et al. [190] performed a study regarding the water content dependent shape memory behavior of P(AAm-*co*-AAc) commercial hydrogel. Authors differentiate two aspects: (i) Shape Change Effect (SCE), which is usually observed in electroactive polymer or piezoelectric materials and (ii) Shape Memory Effect (SME). Both properties were directly related to hydrogel water content. In fact, they found that at low water content hydrogels possessed shape memory effect, whereas at higher water content PAAm-*co*-AAc commercial hydrogel showed both rubber like shape memory effect and water induced shape change. With these findings, they also understand that programming of the system could be performed through the deformation of the hydrogel above Tg or by dehydration after previous deformation of the rubbery-like hydrogel. Such results indicated that the commercial hydrogel PAAm-*co*-AAc could be used as an elastic matrix in a hybrid system to induce the shape memory effect [190]. Following this work, Zhang et al. [191] performed a similar investigation in a double network or semi-interpenetrated network nanocomposite hydrogel composed of PAAm and poly(2-acrylamide-2-methylpropanesulfonic acid). Such hydrogel did not show a remarkable swelling ratio. After the systematic study, authors found that this interesting hydrogel possessed mechano-responsive SCE and water-induced SME properties that are not found in brittle hydrogels with higher swelling ratios [191].

Fan et al. developed a novel dual-responsive shape memory hydrogel of P(AAm-*co*-AAc) with a characteristic thermoplasticity. More concretely, authors prepared hydrogels by simple ternary copolymerization of AAm and AAc with low amounts of a cationic surfactant monomer, in the absence of organic crosslinkers. The resultant hydrogels could exhibit shape memory effects via two different strategies: (i) From an ionic/complex binding between carboxyl groups and ions, which provided additional physical crosslinking points, necessary to lock a temporary shape; and (ii) By salt-strengthened hydrophobic association that enables the formation of a trapped shape. Such physical hydrogel exhibited novel characteristic thermoplasticity, which allowed the change of its permanent shape upon heating and the storage of the shape after cooling, which is a strong step forward compared to the conventional chemically crosslinked shape memory hydrogels [192].

Hu et al. [193,194], also using PAAm as a matrix, fabricated and further optimized [194] a multi-triggered shape memory hydrogel that owns its properties to the presence of two different crosslinkers and stabilizing agents with two different functions: (i) One transforms the shaped hydrogel into an randomly shaped quasi-liquid state; (ii) The other crosslinker, which is present in the quasi-liquid, provides an internal memory that returns the original shaped hydrogel due to the regeneration of the second crosslinker when stimulated. By following the above strategy, authors were able to fabricate two pH sensitive shape memory hydrogels composed of acrylamide chains, crosslinked by duplex DNA and pH-sensitive triplex DNA crosslinking units. The final shape memory hydrogels were indeed stable hydrogels at neutral pH, but dissociated to quasi-liquid states either at acidic or basic pH values. Authors took a step forward and prepared a hybrid system with two different domains, each being selectively triggered to undergo hydrogel/quasi-liquid shape memory dictated transitions [193].

**Figure 9:**
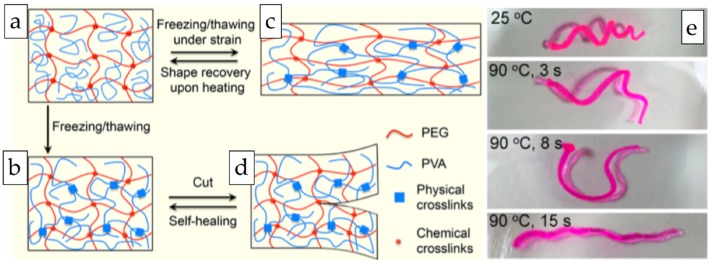
Schematic description of PVA/PEG double-network hydrogels showing both self-shape and self-healing ability (**a**) covalently crosslinked network of PEG mixed with PVA, the “precursor” of the second physical network; (**b**) Using the freezing/thawing treatment, the self-healable double-network hydrogel is produced; (**c**,**d**) the resultant hydrogel gains shape memory effect when the hydrogel is mechanically strong enough to be processed to a deformed state, followed by the formation of the physical network in the hydrogel under deformation; (**e**) photos showing the fast thermally activated shape recovery of the PVA/PEG hydrogel from a temporary twisted shape to the initial straight state. (Adapted with permission from ref. [195]. Copyright 2015 American Chemical Society).

Li et al. [195] were able to develop a chemically crosslinked polymer gel that showed shape memory and self-healing ability. Here, the approach used by the author consists of the development of an interpenetrated network composed of a chemically crosslinked PEG and physically crosslinked PVA hydrogel deriving in a chemical/physical double network (Figure 9a). The PEG/PVA system was subjected to freeze-thawing cycles to obtain the physical entanglement of PVA within the PEG matrix (Figure 9b). By playing with deformations and further freeze-thawing cycles, authors were able to fix a shape after hydrogel deformation (Figure 9c). On removal of the external force, they evaluated the recovery of the hydrogel upon heating [195] (Figure 9e).

By following a similar strategy, Li et al. also developed a PVA-based shape memory hydrogel. In this case, authors fabricated a melamine-enhanced PVA physical hydrogel [196]. They found that the incorporation of a small amount of melamine allow the formation of two type of physical crosslinks in two separates states. In addition to improved mechanical properties and enhanced biocompatibility, such advantage allows the deformation of hydrogel (65% water) and further fixation of the achieved temporary shape. In this case, authors found that heating induced by therapeutic ultrasound was able to trigger the shape memory effect of the hydrogel.

With the aim of obtaining tougher and stronger hydrogel films, Zhang et al. [197] prepared a nanocomposite of CS and GO. Authors applied a water evaporation self-assembly method and a further crosslinking of the solution to obtain a layered CS/GO hydrogel film with bio-inspired nacre-like brick- and mortar-structure, composed of organic and inorganic layers. Besides, the obtained hydrogel showed pH-driven shape memory ability. This interesting feature make this hydrogel film a promising candidate for biomedical applications, such as actuators, biosensors, or even wound dressings. More recently, Ma et al. [198] also inspired by nature, developed a macroscopic anisotropic composite polymeric hydrogel, by the combination of a thermoresponsive GO reinforced PNIPAAm-based hydrogel sheet with a pH-sensitive polyethyleneimine-based fluorescent hydrogel sheet. This combination allowed the fabrication of a bilayer anisotropic hydrogel actuator with on–off switchable and color-tunable fluorescence behaviors. The shape was controllable via temperature changes and the fluorescence color was tunable through pH.

Shape memory hydrogels can also be triggered upon irradiation as recently reported by Huang et al. [199]. In this work, authors fabricated a hybrid (organic-inorganic) shape memory hydrogel (containing more than 76% of water), which comprises of an interpenetrated double network of chemically crosslinked PAAm network and a physically crosslinked gelatin network containing GO. The presence of GO allowed the hydrogel to be triggered upon NIR irradiation, due to its fast and efficient photo-thermal transformation. The thermal-reversible gelatin network was responsible for fixing the temporary shape of the hydrogels, whereas the presence of GO in the chemically crosslinked network permitted the recovery of the permanent shape by irradiation with NIR laser. Authors performed a deep analysis of the effect of the composition in the shape memory effect as well as in the mechanical properties to obtain an optimized composition that could show a stable temporary shape with fast recovery to the permanent shape.

### 4.2. Self-Healing Hydrogels

**Figure 10:**
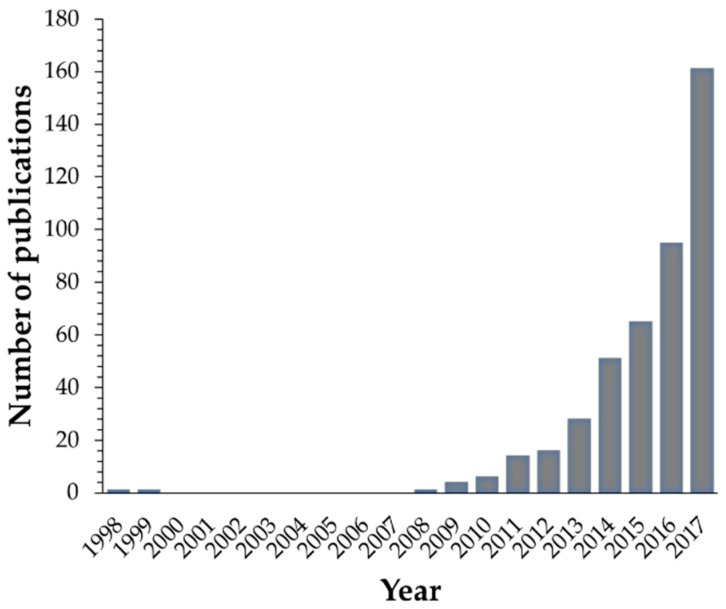
Evolution of publications over the past 20 years on the subject “self-healing hydrogels” (database used for the bibliographic analysis: Scopus^®^ (Elsevier, Amsterdam, The Netherlands consulted on the 3th of May 2018).

Since the last decade, self-healable systems have emerged as a new important class of materials whose origin is the ability to heal or repair itself [200,201]. The interest and vitality of this area of research is highlighted by a nearly exponential increase in the number of publications on the subject over the past 20 years (Figure 10).

This is a complete change in focus from materials that resist environmental damage to responsive, dynamic and smart systems. The development of self-healing polymeric materials has been largely based on mimicking biological healing [202], which is inspired from its innate ability to sense and repair damage in order to restore functionality. Applications of self-healing materials are expected almost entirely in all industries in the future since they effectively enhance the lifetime use of the product and has desirable economic and human safety attributes. The few applications being developed to date are mainly in the automotive, aerospace, and building industries. For instance, self-healing systems are also being adapted for use in paints and coatings, both through the incorporation of micro- and nano-encapsulation of healing agents.

Focusing on polymer hydrogels, the interesting and tunable properties of this functional materials give rise to excellent performances and a wide range of potential applications, as mentioned above. However, such outstanding properties can be lost when the 3D network structure of the gel is damaged (i.e., cracked), resulting mainly in a loss of mechanical properties and eventually in the failure of structure for the desired application. To avoid the consequences of this event, huge efforts have been made in the past years to develop polymer gels with the ability to self-repair or self-heal and thus restore their initial structure and functionalities. The approaches used for the development of self-healable hydrogels are mainly based on the constitutional dynamic chemistry (CDC) concept, key factors of which are dynamicity and reversibility, involving covalent (chemical self-healing gels) and non-covalent chemistry (physical self-healing gels) [203,204]. Self-healing gels based in this CDC concept can recover their original shape and restore their initial properties, in multiple cycles, due to the reversibility of the process. In both cases, the presence of functional groups able to perform such physical or chemical dynamic interactions, is necessary for self-healing purposes [102,200,205]. The advantage that gels present over polymers for self-healing is noteworthy, since the presence of the solvent enables the formation of a mobile structure around the damage area, which could eventually facilitate the healing.

Phadke et al. [206] reported for the first time a rapid self-healable hydrogel, in which the repair occurred within seconds of the damage or breakage into two hydrogel pieces (glue of two parts) and in an aqueous environment. The prepared hydrogel presented reversible healing, externally controllable by pH changes, with multiple cycles of healing-separation without affecting mechanical properties and repair kinetics. To induce such ability, they decorated the polymer hydrogel (chemical gel) prepared from acryloyl-6-aminocaproic acid precursors with dangling hydrocarbon side chains containing polar functional groups that would induce hydrogen bonds responsible for the polymer healing. At low pH, carboxyl groups are mostly protonated, which allows them to form hydrogen bonds with other carboxyl groups or amide groups, resulting in the repair of the hydrogel. However, at pH > pKa of the precursor monomer, carboxyl groups of the polymer are protonated, inhibiting the hydrogen bond formation and thus, the healing of the material. In addition, authors found that the side chain length was affecting the healing ability of the system. Short side chains do not favor hydrogen bond interactions among functional groups due to the limited availability of the carboxyl groups. In contrast, when the side chains exceed in length, they begin to front a larger steric hindrance to the interactions between the carboxyl and amide groups. This result highlighted the importance that the balance of hydrophilic-hydrophobic interaction has in hydrogels to present a self-healing property.

Along these lines, Tuncaboylu et al. [207,208,209] developed a self-healing hydrogel by the micellar free-radical polymerization of hydrophilic AAm in the presence of long alkyl chains of hydrophobic monomers. Interaction between hydrophobic blocks avoids dissolution in water and the dynamic nature of the junction points between the network chains (non-associated hydrophobic blocks) provides a self-healing efficiency to elongation at break of about 100% together with a high degree of toughness [207,208]. Following this trend, Gulyuz et al. [210] prepared self-healing physical PAAc hydrogels by micellar copolymerization of the AAc with an alkyl monomer (stearyl methacrylate, C18) in a surfactant micellar solution (sodium dodecyl sulfate). Authors found that by replacing AAm with AAc monomer, the obtained hydrogel results in stronger self-healing due to the cooperative hydrogen bonds formed between the carboxyl groups stabilizing the hydrophobic domains. In addition to the self-healing property, the obtained hydrogels exhibit high modulus (6–53 kPa), high fracture stress (41–173 kPa) and high elongation ratios at break (1800–5000%).

Zhang et al. [211] worked with the well-known PVA physical hydrogel (prepared using the freezing/thawing method) and found that it can autonomously self-heal at room temperature without the need for any stimulus or healing agent. The obtained self-healable hydrogel was mechanically strong, showing high fracture stress. The autonomous self-healing ability of the PVA hydrogel was caused by the formation of hydrogen bonding between the PVA chains. In fact, the authors indicated that the key factor to obtaining fast and efficient self-healing of PVA hydrogel was the balance between a sufficient amount of free hydroxyl groups of PVA on cut surfaces required to form interchain H-bonds and enough chain mobility ensuring chain diffusion across the interface.

As indicated in previous sections of this review, thermoresponsive polymer PNIPAAm played an important role in the field of polymer gels due to the numerous stimuli-responsive hydrogels and microgels developed involving this polymer. However, approaches to develop PNIPAAm and PNIPAAm-based hydrogels with self-healing abilities are still scarce due to the side groups of PNIPAAm that hinder secondary interactions. Along these lines, Gulyuz et al. [212] once again took advantage of the micellar radical copolymerization and presented for the first time the preparation of PNIPAAm hydrogels with self-healing property. In fact, the hydrogels have autonomous self-healing ability with an efficiency of up to 100%, as demonstrated by mechanical measurements. However, the obtained hydrogel loses its self-healing ability upon swelling due to micellar structure disintegration that increases hydrophobic interactions. Authors overcomed this problem by the incorporation of AAc (replacing some NIPAAm monomers) that binds with the surfactant so that it remains limited in the polymer. The addition of AAc also contributes to the improvement of mechanical properties.

The evolution on hydrogel research has brought about the development of materials that exhibit improved mechanical properties. However, most of the reported hydrogels that exhibit self-healing properties are in general mechanically weak. To overcome this problem, several authors hybridized self-healable hydrogels with GO, similarly to what was also observed in the previous section regarding shape memory hydrogels [213,214,215]. In this respect, Liu et al. [216] proposed the preparation of chemical self-healing PAAm hydrogel synthesis in the presence of GO. Authors suggested that the formation of hydrogen bonds between the PAAm polymer chains and the GO sheets were a determinant for self-healable hydrogels to present strong mechanical properties [217]. Such strong interactions between PAAm and GO, being difficult to be broken, gave stability to the hydrogel during swelling and deformation (application of a load). GO sheets have been used as fillers in polymer composites, improving the mechanical properties of polymer substrates. However, the use of GO sheets assembled into hydrogels can act not only as a crosslinking agent but also contribute to the self-healing ability through the dissipation of crack energy [213].

At the beginning of this section, we have indicated that the ability of self-repair is desired property in fieldls like aeronautics, coatings, paintings, etc. However, to confer the self-healing ability into smart hydrogels can also be useful in biomedical applications. For instance, when considering smart hydrogels to be used as implants in the body, it should be noted that these implants are under constant movement and thus at risk of mechanical damage. As a consequence, this fact could directly affect the functionality of the implant or even be the cause of an infection. In this regard, as stated by Li et al., developing a hydrogel able to self-heal could contribute to extending the application of the hydrogel and the lifetime of the material. These authors developed a mussel-inspired self-healing injectable hydrogel with anti-biofouling and anti-microbial properties [218,219,220]. For the development of such self-healing hydrogels, the author took into consideration the self-repair ability of mussel’s byssal threads, which is mainly attributed to the reversible metal-catechol coordination (catechol group of an amino acid). In the work of Li et al., authors based the hydrogel preparation on the self-assembly of a ABA tri-block copolymer. As block A, authors used catechol-functionalized PNIPAAm as the thermosensitive block and as hydrophilic block B of the copolymer PEO. The temperature sensitivity provided by PNIPAAm-based block, A, confers control over the liquid or solid-like state of the copolymer. For instance, at temperatures below a critical value, the hydrogel presents a liquid-like behavior, but when heating above certain temperatures, the gelification and hence, hydrogel formation occurs. Rheological studies to determine the self-healing ability of the material as well as visual evidences demonstrated that the hydrogel can heal after being damaged within seconds. Authors attributed the self-healing mechanism to catechol-mediated hydrogen bonding.

The mussel-inspired strategy and chemistry was also followed by other authors. For instance, Hou et al. [221] developed supramolecular stimuli-responsive fast self-healing hydrogels, with the particularity of being highly stretchable depending on composition and deformation conditions. Compared to covalent bonds, supramolecular interactions are reversible and can respond to different external stimuli as pH, temperature, and external force, a property that imparts dynamic features and hence, self-healing capacity to this kind of hydrogels. In this case, the obtained hydrogel based in a monomer bearing a catechol group, can be cast and photo-crosslinked into a desire shape in just one step. For the same, authors prepared supramolecular hydrogels using photopolymerizable precoordinated catechol−Fe^3+^ complexes as the multifunctional cross-linkers and AAm as the monomer. The self-healing ability of the obtained supramolecular hydrogel follows a dynamic process due to the re-association capacity of precoordinated metal−catechol interactions.

### 4.3. Stimuli-Responsive Self-Healing Microgels

In this field of self-healing polymer hydrogels, it is of importance to highlight the growing interest regarding the use of polymeric microgel and nanogel particles in the development of autonomous self-healing systems [222,223]. The ease of synthesizing microgels to display a wide range of stimuli-responsiveness and mechanical properties at the particle level and in assemblies provide microgel-based materials with real potential for self-healing applications.

Latnikova et al. [222] proposed a new approach towards ‘‘active’’ self-healing coatings with the use of microgels as reservoirs of “Green” corrosion inhibitors. In this case, microgels act as reinforcement while retaining the healing agent to be delivered when coating is damaged. Lyon et al. went a step ahead and used microgels as building blocks for self-healing materials, not as carriers or capsules. The authors were able to fabricate a self-healing hydrogel composed of microgel of P((NIPAAm-AAc) by means of a layer-by-layer deposition technique over a polycation. They built a 3D network (film) upon the self-assembly of microgel layers sustained by the different surfaces charges among microgels and polycation surfaces [224,225,226,227]. The peculiarity of this system is based on the dynamics that microgels have within the layers. When a damage or crack is generated in the hydrogel film, there is a lack of equilibrium in terms of electrical charges. When the system is solvated, this allows the reconfiguration of microgels toward the less energetic state, which entails the healing of the damage.

## 5. Conclusions

In the field of materials science and engineering, the design, research and development of new and improved smart structures and systems is currently a hot topic. In this context, functional stimuli-responsive gels are of great interest due to their versatility and possible application in fields as diverse as biomedicine, catalysis, or even biosensors.

The relevance of the subject dealt in this review is clearly reflected in the considerable number of relevant publications. In this review, we intended to cover the most recent and high-impact publications using functional stimuli-responsive gels. The review started from macro to micro, i.e., from hydrogels to microgels, ending with a new generation of functional gels. Stimuli-responsive functional hydrogels and microgels are used for a broad range of applications, and several publications can be found in literature. PNIPAAm is the most used thermoresponsive polymer. In many cases, PNIPAAm is combined with another polymer to add a new functionality to the hydrogel. In microgels, PNIPAAm is most commonly combined with an inorganic component, ranging from metallic nanoparticles to carbonaceous particles. In the last part of the review, the new generation of functional stimuli-responsive gels, able to self-repair or/and possess shape memory property, are described. Among others, PAAm, PVA and PEG are the commonly used polymers in this new generation of functional gels. The development of Shape Memory Polymers suitable for biological media in a living system is a still a challenge today. In this regard, shape memory hydrogels that are predominantly water stand out as alternative candidates for biomedical applications due to the use of water as a driving force for the desired shape memory effect. The advantages and attributes of functional gels (macro and micro) have been highlighted throughout the entire review, but the most promising is the ability to self-heal, expected in almost all industries in the near future. Self-healable hydrogels go through improvements in their mechanical properties without affecting their intrinsic properties, and importantly, their responsiveness to external stimuli. 

In summary, this review demonstrates the prominent role of smart functional gels in materials science; its versatility and tunable properties allows usage in a wide range of applications.
